# Assessment of the therapeutic value of new medicines marketed in Australia

**DOI:** 10.1186/2052-3211-6-7

**Published:** 2013-09-10

**Authors:** Michael J Wonder

**Affiliations:** 1 Wonder Drug Consulting, Sydney, NSW, Australia

## 

I read with interest the study by Vitry et al. on the therapeutic value of new medicines approved for marketing in Australia in the mid 2000s using two European classification systems and their finding that “only a minority of the new medicines in Australia provide added therapeutic value compared to existing treatments” [1].

They seem to be confused about the definition of economics and differences between ‘new’ and ‘innovation’ and ‘benefit’ and ‘value’.

According to Vitry et al., “from an economic perspective, pharmaceutical products are considered innovative as long as they are new and the success of innovation is defined in terms of sales, with the assumption that higher sales is a measure of the intrinsic worth of the innovation.” Economics is the science that deals with the production, distribution, and consumption of goods and services; in other words, the study of inputs (resources) and outputs (outcomes) [2]. In the context of health care, economics is the study of the (health) benefits of a given intervention (new or old) and its related costs. The definition does not encompass aspects of novelty, innovation and sales. Their statement is more befitting of a pharmaceutical industry perspective.

Furthermore, Vitry et al. note “between 2000 and 2009, of the 984 new medicines or new indications approved in France, more than half did not provide anything new.” This statement is confusing; perhaps they meant to say “nothing of therapeutic benefit/gain.”

The magnitude of a medicine’s incremental health gain (i.e. therapeutic benefit) over current treatment combined with the level of unmet clinical need for effective interventions for patients with the disease/condition yields an estimate of its therapeutic value. Such determinations are not straightforward when there is an efficacy gain and a safety loss; such assessments are currently made on a case-by-case basis using expert value judgment. In diseases/conditions where there are many treatment options, a new medicine might be therapeutically superior to some treatments and similar or even inferior to others. A new medicine might be therapeutically superior to a low dose of a given medicine but clinically inferior to a higher dose of the same medicine. Vitry et al. are rather vague on what comparisons they think should be made for a new medicine.

The determination of a new medicine’s therapeutic benefit and value currently lies in the realm of the HTA agencies such as the PBAC than in the realm of the regulators such as the TGA. Vitry et al. claim that this is a role for the TGA; “another limitation of this study is the lack of gold standard methodology for the evaluation of the therapeutic value of new medicines. Regulatory agencies do not currently use standardized processes but mostly rely on expert judgment.” The current role of the TGA does not include the determination a new medicine’s therapeutic value. I am not aware that the TGA is seeking to usurp the role of the PBAC.

It should come as no surprise that the TGA approves new medicines deemed by HTA agencies to be of modest or similar therapeutic benefit/value. The TGA acts like other medicine regulators in this regard. Vitry et al. seem to imply that the TGA should only register new medicines that are associated with a clear therapeutic benefit; they fail to consider the consequences of having fewer medicines on the market to the degree that only one medicine might be available in a given pharmacological/chemical class (e.g. only one statin). The more important issue is whether payers pay more for new medicines that do not provide any additional therapeutic benefit. There is ample local experience to indicate that this has not occurred.

Vitry et al. state that “stricter regulatory approval criteria would ensure better safety of the public”. They do not cite any examples where the safety of the Australian public has been compromised as a result of supposedly lax approval criteria by the TGA. The use of stricter criteria will result in the approval of fewer new medicines in Australia.

Vitry et al. also state that the use of “stricter regulatory approval criteria would…and simplify the reimbursement process” but they provide no details on what PBS processes they refer to.

Finally, the authors claim that the public, health care professionals and policy makers have a belief that all new medicines bring a “therapeutic innovation and better health outcomes” and that the “use of a simple classification system could provide a useful, simple and transparent way to better inform the public and health professionals on the therapeutic value of new medicines.” Their claim that the public, health care professionals and policy makers think that all new medicines are associated with therapeutic value is supported by a reference that reports on a case study for single new medicine in the UK. They present no solid evidence to indicate that such beliefs are widespread in Australia.

## Competing interest

The author declares that he has no competing interests.
